# Physicochemical Characterization of Ca- and Cu-Decorated TiO_2_ Microparticles and Investigation of Their Antimicrobial Properties

**DOI:** 10.3390/ma17184483

**Published:** 2024-09-12

**Authors:** Andreea Neacsu, Viorel Chihaia, Razvan Bucuresteanu, Anton Ficai, Roxana Doina Trusca, Vasile-Adrian Surdu, Adela Nicolaev, Bogdan Cojocaru, Monica Ionita, Ioan Calinescu, Viorica Parvulescu, Lia-Mara Ditu

**Affiliations:** 1 Institute of Physical Chemistry “Ilie Murgulescu”, Romanian Academy, Splaiul Independentei 202, 060021 Bucharest, Romania; vpirvulescu@icf.ro; 2 Microbiology Department, Faculty of Biology, University of Bucharest, Intr. Portocalelor 1–3, 060101 Bucharest, Romania; razvan.bucuresteanu@drd.unibuc.ro (R.B.); lia-mara.ditu@bio.unibuc.ro (L.-M.D.); 3 Faculty of Chemical Engineering and Biotechnologies, University Politehnica of Bucharest, 060042 Bucharest, Romania; anton.ficai@upb.ro; 4 National Centre for Micro and Nanomaterials and National Centre for Food Safety, Faculty of Chemical Engineering and Biotechnologies, University Politehnica of Bucharest, Spl. Indendentei 313, 060042 Bucharest, Romania; truscaroxana@yahoo.com; 5 National Research Centre for Micro and Nanomaterials, Faculty of Applied Chemistry and Materials Science, University Politehnica of Bucharest, Polizu Street No. 1–7, 011061 Bucharest, Romania; adrian.surdu@upb.ro; 6 National Institute of Materials Physics, 405A Atomistilor Street, 077125 Magurele, Romania; adela.nicolaev@infim.ro; 7 Department of Inorganic Chemistry, Organic Chemistry, Biochemistry and Catalysis, Faculty of Chemistry, University of Bucharest, 4–12 Regina Elisabeta Av., 030018 Bucharest, Romania; bogdan.cojocaru@chimie.unibuc.ro; 8 Faculty of Chemical Engineering and Biotechnologies, University Politehnica of Bucharest, Polizu Street No. 1–7, 011061 Bucharest, Romania; ionita_monica@yahoo.com; 9 Faculty of Applied Chemistry and Materials Science, University Politehnica of Bucharest, Polizu Street No. 1–7, 011061 Bucharest, Romania; ioan.calinescu@upb.ro

**Keywords:** antibacterial, zeta potential, Ca-Cu composite, rutile, decorated

## Abstract

Ca- and Cu-decorated TiO_2_ microparticles are titanium dioxide nanoparticles that have been decorated with calcium and copper ions. TiO_2_, CaO, and CuO are low-cost, non-toxic, and non-hazardous materials. The aim of the present study was the physicochemical characterization of Ca- and Cu-decorated TiO_2_ microparticles and the evaluation of their antimicrobial activity. Thus, Ca^2+^ and Cu^2+^ species were incorporated onto TiO_2_ surfaces by a two-step wet method. The obtained TiO_2_-CaO-CuO composites were characterized by several experimental techniques. The electronic structure and charge properties of the composites were investigated by density functional theory calculations. Furthermore, the composites were successfully tested for inhibitory effects on *Staphylococcus aureus*, *Pseudomonas aeruginosa*, *Escherichia coli*, and *Candida albicans* standard strains. The zeta potential data indicate that the physiological condition of investigated microbial strains was strongly affected in presence of a dispersion of 10 μg/L of composites in a saline phosphate buffer also, the recorded SEM images show a damaged microbial cell surface in the presence of composites.

## 1. Introduction

The combination of TiO_2_ with CaO and CuO has various applications and uses across different fields, like ceramics, paints, and coatings. The combination of TiO_2_ with CaO and CuO can result in the production of compounds such as calcium copper titanate (CaCu_3_Ti_4_O_12_—which is a standalone compound) or CaCO_3_-TiO_2_-CuO/Cu_2_O types of pigments, with variable proportions of constituent elements [1,2]. Rutile titanium dioxide nanoparticles have been modified or decorated with calcium and copper ions or nanoparticles in order to obtain pigments exhibiting enhanced antibacterial properties [3,4].

CaO is a key material in the preparation of CaCO_3_-TiO_2_ composite particles. The preparation of CaCO_3_-TiO_2_ composites can be achieved through various methods that involve the grinding or milling of particles to reduce their size, increase the surface area, and promote the reaction between the reactants [5]. In a practical way, the mechanochemical method for CaCO_3_-TiO_2_ synthesis is similar to the wet grinding method in terms of particle grinding and activation, but they differ in the use of a liquid medium [6,7,8]. Due to the presence of TiO_2_ in the composition, CaCO_3_-TiO_2_ composites exhibit similar pigment properties to pure TiO_2_, and they also inherit its antibacterial properties. When coating TiO_2_ particles on the surfaces of CaCO_3_ particles, the composite pigment increases the utilization efficiency of TiO_2_ and reduces the consumption of pure TiO_2_. This helps to lower the production costs of the composite while maintaining its pigment properties [6,9]. Furthermore, CaCO_3_-TiO_2_ composites are generally unstable in acidic media due to the poor acid-fastness of CaCO_3_. This limits their applications in acidic environments [6]. Therefore, CaCO_3_-TiO_2_ composite particles are most commonly used as pigments, but their applications may be limited to specific industries, such as paint and coating, and they may not be suitable for all uses [10].

CaO is largely used in the preparation of cement-based materials and plays a significant role in the hydration process of cement and the development of its internal structure. The addition of TiO_2_ nanoparticles to CaO in portlandite in the presence of other minerals has been reported to improve cement durability, reduce capillary porosity, and increase the density of the cement matrix [11]. TiO_2_ can accelerate the early hydration reaction of cement pastes, which is important for the development of the mechanical properties of cement, while, at later hydration times, TiO_2_ nanoparticles may hinder hydration and reduce the ability of water molecules to access poor hydrated cement grains [12]. Additionally, TiO_2_ nanoparticles confer photocatalytic properties to portlandite cements, making them self-cleaning and capable of decomposing or deactivating volatile organic compounds and removing bacteria.

Further, TiO_2_-coated CuO composites are important due to the synergistic properties and benefits they offer in various applications. CuO-TiO_2_ composites can serve as multifunctional fillers in coatings, providing both corrosion protection and antibacterial properties simultaneously. This multifunctionality is advantageous in applications where both properties are desired, such as in marine coatings or medical devices [13,14]. When CuO is incorporated into TiO_2_, it can enhance light absorption and extend the absorption wavelength range to visible light. Cu acts as a co-catalyst, facilitating the transfer of electrons between TiO_2_ particles and the reaction species, thus promoting photocatalytic reactions [15,16]. In addition to photocatalytic properties, Cu can also improve the stability and durability of TiO_2_ composites, but the specific effects may vary depending on the concentration of CuO and the method of incorporation [17,18].

Ca-decorated TiO_2_ composite particles have similar pigment properties to pure TiO_2_. This suggests that copper could potentially bind to the composite particles through the TiO_2_ component, just as it binds to TiO_2_ alone. It becomes a challenge to add Cu in such composites in order to improve the existing properties of the pigment and allow for the cost-effective utilization of TiO_2_. Cu forms strong interactions with the surfaces of TiO_2_ particles, and this leads to the improved stability of the composite material, making it more resistant to degradation and maintaining its performance over a longer period of time.

This article is a continuation of our work related to Ca- and Cu-decorated TiO_2_ microparticle composites [3]. As in the previously published work, this study presents new decorated composites, in which the percentage of calcium has been changed. Starting from a derivative synthesis method, the new pigments were enriched with sodium in order to gain superior antibacterial properties compared to previously synthesized pigments. The alkaline conditions provided by the hydroxides facilitate the formation, stability, and deposition of TiO_2_ onto the carbonate substrate, resulting in composite nanoparticles with desirable properties for various applications. On the other hand, when adding excess alkaline hydroxides, such as Ca(OH)_2_ and NaOH, the formation of a uniform coating of TiO_2_ particles can be disrupted. To overcome this inconvenience and to improve the properties of the pigment, CuO was added in the synthesis. These new composites were studied from a structural point of view, and the antibacterial properties under darkness conditions were verified. The purpose of these investigations is to develop future products for medical supplies and health-care facilities, in the composition of which the materials synthesized by us should be included.

## 2. Materials and Methods

### 2.1. Materials

For the synthesized composites, S1 and S2, a mechanochemical was used [3]. A titanium dioxide TYTANPOL type was used as pigment in preparations of the samples S1 and S2. Any other substance used in preparations meets the quality and purity standards provided by Sigma-Aldrich company (St. Louis, MO, USA). The presence of sodium in the samples is due to the preparation in an alkaline environment and is related to the pH adjustment. The sample names and abbreviations and the corresponding quantities of Ca and Cu are depicted in Table 1.

### 2.2. Methods

Scanning electron microscopy (SEM): the electron microscopy images were obtained using a Quanta Inspect F50 (FEI Company, Eindhoven, The Netherlands) equipped with a field emission gun (FEG) with a 1.2 nm resolution and an energy-dispersive X-ray spectrometer (EDX) with a MnK resolution of 133 eV K.

X-ray diffraction (XRD) analysis: powder XRD patterns were collected with a Shimadzu XRD-7000 (Kyoto, Japan) diffractometer employing CuKα radiation (λ = 1.5418 Å), operating at 40 kV and with a current intensity of 40 mA in the 2θ range of 5–80°.

X-ray photoelectron spectroscopy (XPS) analysis: The X-ray photoelectron spectroscopy (XPS) analysis of the sample was performed in an AXIS Ultra DLD (Kratos Surface Analysis) setup using Al Kα1 (1486.74 eV) radiation produced by a monochromatized X-ray source at operating power of 144 W (12 kV × 12 mA). The base pressure in the analysis chamber was around 1 × 10^−9^ mbar.

UV-Vis spectroscopy: the UV-Vis absorption spectra of the modified TiO_2_ samples were obtained in diffuse reflectance (DRS) mode using a JASCO V570 spectrophotometer (Tokyo, Japan).

Fourier-transform infrared spectroscopy (FT-IR): FT-IR spectral data of solid powders of S1, S2 and TiO_2_ were recorded at room temperature by Nicolet iS10 FT-IR Spectrometer (Thermo Scientific, in Waltham, MA, USA) covering the range of 4000 to 600 cm^−1^. The spectra were acquired with an average of 32 scans with spectral resolution of 4 cm^−1^ in attenuated total reflectance (ATR) mode.

Density functional theory calculations: We consider the adsorption of Ca and Cu atoms on the surface (001) of the rutile titanium oxide, which is a less stable and more reactive surface compared with other low-index surfaces of rutile TiO_2_. In order to understand the trend of interaction of the calcium and copper with the rutile surfaces, we performed density functional theory (DFT) calculations by the quantum solid state code CASTEP [19], with a high-accuracy calculation scheme (on-the-fly generated ultrasoft pseudopotential, exchange-correlation functional Perdew Burke Ernzerhof, energy cutoff of 490 eV, Brillouin zone discretized by Monkhorst–Pack scheme, with a spacing of 0.04 Å^−1^, TS semi-empirical dispersion correction, [20]) that reproduced the experimental lattice parameters of rutile TiO_2_ (*a* = *b* = 4.5937 vs. 4.5936 Å, and *c* = 2.9586 vs. 2.9587 Å, values from present calculations and experiment, respectively, [21]) well. We considered the spin-polarized wave functions as the investigated systems with copper atoms that might have magnetic properties.

**Figure 1:**
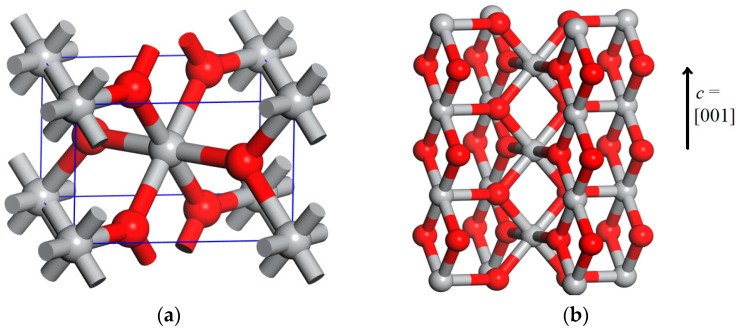
The structure of the bulk primitive cell of rutile TiO_2_ (**a**) and the column-like arrangement of the Ti-O-Ti-O rhombs along the *c*-axis (**b**). The gray and red spheres represent the titanium and oxygen atoms, respectively.

The rutile polymorph of the titanium oxide belongs to the P42/mnm space group and has six atoms per unit cell: two titanium atoms arranged in an octahedral coordination to six oxygen atoms, which are bonded to three titanium atoms, as shown in Figure 1a. The oxygen and titanium atoms form columns of rhomboidal units, Ti-O-Ti-O, aligned along the *c*-axis (see Figure 1b). The neighbor columns are orthogonal to each other and are connected two by two by Ti-O bonds that are orthogonal to the *c*-direction.

**Figure 2:**
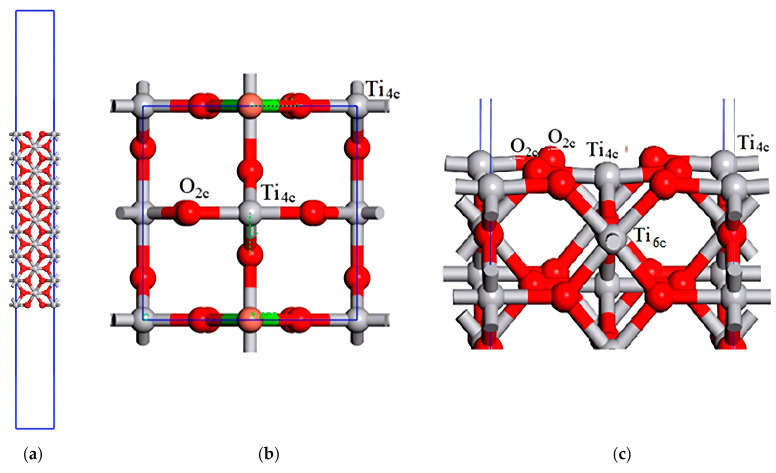
The simulation cell used for the slab calculations, representing the surface of rutile TiO_2_(001) (**a**), top (**b**), and side (**c**) views of the supercell used for the adsorption study (for simplicity, only four layers are shown). The adsorption sites on the surface of rutile TiO_2_(001) are indicated: O_2c_/Ti_4c_—atop two/four coordinated oxygen/titanium atoms from the top layer; Ti_6c_—the titanium atom from the second layer, bonded to two O_2c_ atoms from the top layer. The color significations are the same as in Figure 1.

The surface TiO_2_(001) is represented by a slab of seven atomic layers that assure the convergence of the adsorption energy for the atoms of Ca and Cu. Along the direction *c* // [001], a large void space of 30 Å width is inserted in order to eliminate the interaction between the successive slabs, built by the 3D periodic boundary condition (see Figure 2a). The columns of Ti-O-Ti-O rhombs are interrupted by cleavage of the (001) surface. Thus, the three- and six-coordinated atoms of oxygen and titanium, respectively, become two- and four-coordinated on the surface top layers, and they are denoted as O_2c_ and Ti_4c_. The surface exposes a chain of intermittent trimers, O_2c_-Ti_4c_-O_2c_, oriented along the directions [100] or [010], depending where the cleavage plane is applied on the successive atomic layers (Figure 2b). The atoms on next layers have a bulk-like coordination (Figure 2c).

The calculation scheme for the surface is the same as in the case of the bulk calculations, but with a k-grid 3 × 3 × 1 instead of 3 × 3 × 5, as was considered for bulk. In order to avoid direct interaction between the adsorbates located in periodic neighbor cells, a supercell determined by the transformation matrix $\left(\begin{array}{ccc} 1 & 1 & 0\\ -1 & 0 & 0\\ 0 & 0 & 1 \end{array}\right)$ was applied to the primitive cell of TiO_2_ (see Figure 2b,c). In order to exclude mutual interaction between the adsorbates and their periodic images, a larger supercell would have been necessary, but the increase in the supercell size would have made impractical calculations that involve the adsorbate and top-layer relaxation. The atoms on first two layers on face on which the Ca and Cu atoms are adsorbed are completely relaxed, and the other atoms from the slab are frozen to the position corresponding to the bulk system. The geometry optimization is performed by the two-point steepest descent method (TPSD), included in *CASTEP* code. In all the investigated slab systems, the lattice parameters are kept fixed to the corresponding bulk values. The maximum ionic force tolerance limit was set to 0.01 eV/Å. Several adsorption sites can be considered for M = Ca and Cu atoms. The most stable adsorption was with the metal atom over the two non-bridged O_2c_ atoms from two successive triplets, O_2c_-Ti_4c_-O_2c_ (see Figure 2b,c). For simplicity, we call this adsorption site T_6c_, as the adsorption is over a T_6c_ from the second atomic layer. The adsorption energies per atom were calculated by the following equation:

$$E_{ads/atm}=\left[E\left({Ca}_{x}{Cu}_{y}/{TiO}_{2}(001)\right)-\left(E\left({TiO}_{2}(001)\right)+xE\left(Ca\right)+yE(Cu)\right)\right]$$

where $E\left({TiO}_{2}(001)\right)$ and $E\left({Ca}_{x}{Cu}_{y}/{TiO}_{2}(001)\right)$ are the energies of the clean slab and of the slab with *x*,*y* = 0 or 1 adsorbed atoms of Ca and Cu, respectively. $E\left(X\right)$ is the energy of an isolated atom of each chemical species, *X* = Ca or Cu, which is calculated for one atom of type *X* placed in a big, empty cubic box with an edge length of 30 Å. The used DFT calculation scheme for an atom is the same as for the other systems, except that the grid from the reciprocal space is reduced to the *Γ* point.

Biological test: The antimicrobial assays were performed using the following standard microbial strains from microbial collection of University of Bucharest, Faculty of Biology, Microbiology Department: *Staphylococcus aureus* ATCC 25923, *Escherichia coli* ATCC 25922, *Pseudomonas aeruginosa* ATCC 27853 and *Candida albicans* ATCC 10231. Two successive passages on appropriate nutritious agar medium were performed before starting the experiment.

For the qualitative screening of the antimicrobial properties of two tested samples, microbial suspensions with standard densities of 1.5 × 10^8^ CFU/mL (for bacterial strains) and 6 × 10^8^ CFU/mL (for yeast strain) were used. All the tests were performed according to the adapted spot diffusion method (Clinical Laboratory Standard Institute, 2023). Microbial inoculums were seeded on the specific agar medium (Muller Hinton agar for bacterial strains and Sabouraud agar for yeast strain), and an amount of approximately 20 µL of each sample (100 mg/mL tested concentration) was spotted over. The plates were left at room temperature for diffusion and then incubated at 37 °C for 24 h. The sensibility of tested microbial strains was evaluated by measuring the diameters of the inhibition zones that appeared under and around the sample spots, expressed in millimeters (mm). The experiment was carried out on three different occasions, and the results were expressed as the average of the three obtained values.

Quantitative assessment of the capacity of tested samples to inhibit the microbial growth was performed using serial microdilution method. The MIC (minimal inhibitory concentration) values of the tested samples were determined using the serial microdilution method performed in nutritive broth, which was added in sterile 96-well plates. For each tested sample, binary dilutions were performed, covering a range of concentrations between 10 mg/mL and 19.5 µg/mL. Further, 15 μL of microbial suspension adjusted to 1.5 × 10^7^ CFU/mL were added in each well, and the plates were incubated at 37 °C for 24 h. The negative control (C−) was considered the sterile liquid medium, and the positive control (C+) was broth medium inoculated with microbial suspensions. Since the tested pigments formed a white deposit at the base of the wells, the MIC values were established by visual analysis of the wells and comparing with the C+ and C−, but also by recultivating the microbial suspension from the corresponding MIC well, to confirm the inhibitory effect on microbial growth and multiplication. The VCC (viable cell count) method was used in order to determinate the CFU/mL values, after serial tenfold microdilutions and inoculation in triplicate on agar medium. The VCC results were statistically analyzed using the GraphPad Prism v9 program, developed by GraphPad Software, San Diego, CA, USA. All experiments were performed in three independent determinations.

Dynamic Light Scattering (DLS) measurements: The zeta potential (ZP or ζ) values of the formulated nanoemulsions and bacterial solutions were studied by dynamic light scattering (DLS) using Malvern Zetasizer Nano-ZS equipment (Malvern Instruments, Malvern, UK). The sample formulations were equilibrated for 3 min prior to the measurements. The data records were performed at 37 °C, considering the properties of phosphate-buffered saline (PBS) used in solution preparations, standard viscosity of 0.785 cP, refractive index of 1.34 and dielectric constant of 78.3.

## 3. Results

### 3.1. Scanning Electron Microscopy

**Figure 3:**
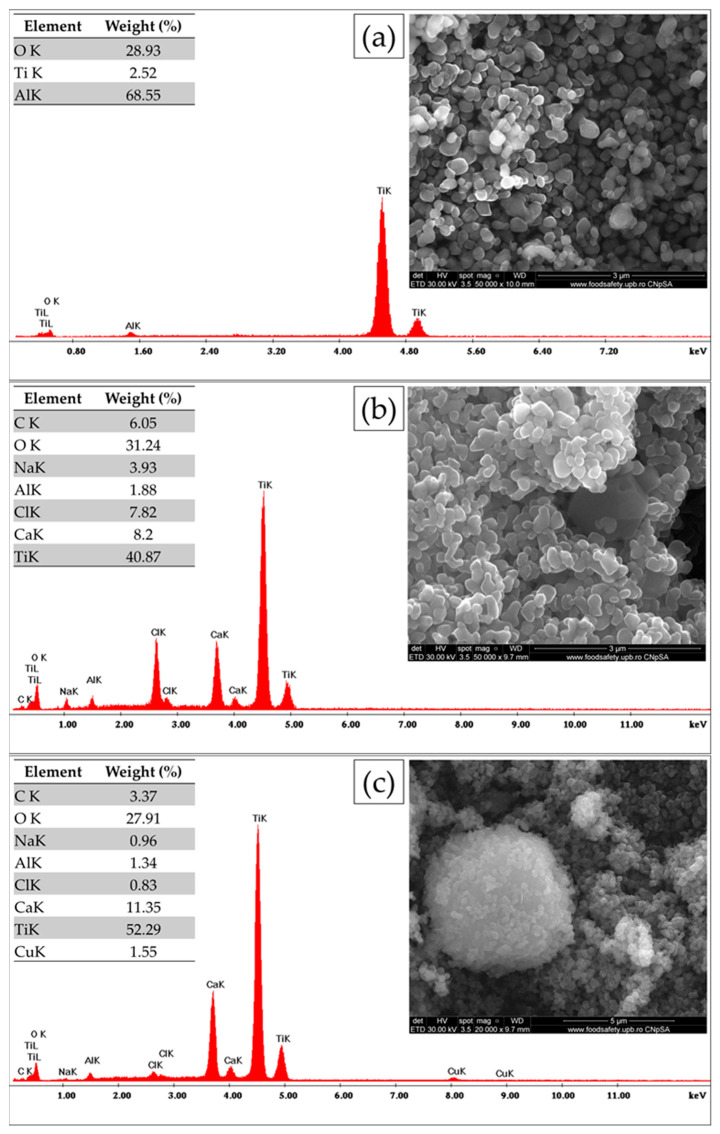
Scanning electron micrograph (SEM) and corresponding SEM-EDX spectra with the weight percentage of elements obtained for rutile TiO_2_(**a**), sample S1 (**b**), and sample S2 (**c**).

The morphological and compositional changes to the Ca- and Cu-decorated TiO_2_ particles are presented in Figure 3. The EDX analysis showed the presence of the main elements in the pigment powder, including carbon identification. The presence of carbon is due to the possible absorption of carbon dioxide into the samples. In Figure 3a, the surface morphology of the round nanoparticles of the rutile are presented. The SEM images of samples S1 and S2 (Figure 3b,c) revealed that the pigments’ core consisted in large hexagonal particles of hydroxides, which were densely decorated by TiO_2_ and carbonate. This may have been a consequence of the agglomeration of the TiO_2_ during the activation process and of the ongoing carbonation of the hydroxide crystals.

### 3.2. X-ray Diffraction

**Figure 4:**
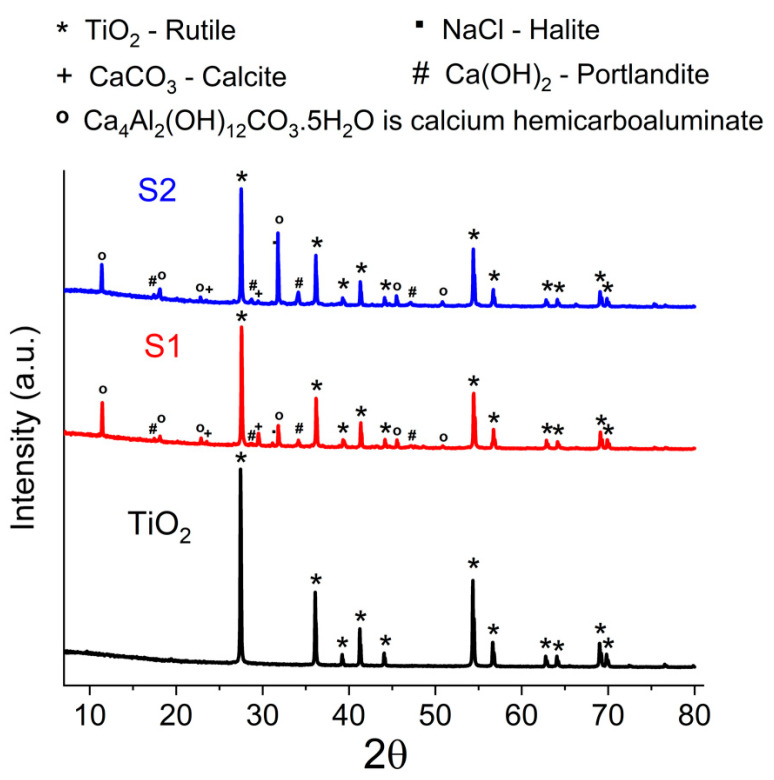
X-ray diffraction patterns of S1, S2, and TiO_2_ samples.

The X-ray diffraction (XRD) patterns of the TiO_2_ support, S1, and S2 samples are presented in Figure 4. All the samples exhibit diffraction peaks at 27. 43°, 36.08°, 39.18°, 41.24°, 44.04°, 54.32°, 56.62°, 62.77°, 64.04°, 69.00°, and 69.81°, which are attributable to the tetragonal phase of the rutile TiO_2_ (JCPDS PDF card 00-021-1276) [22]. Similarly, the S1 and S2 composites with different CaO and CuO contents also exhibit the characteristic diffraction peaks of the rutile TiO_2_ phase. Although sample S2 contained oxide species of copper, in the X-ray diffractogram, no characteristic peaks were observed. This is the result of both their low concentration and the possibility of their masking by the characteristic peaks of other species that were in higher concentrations. Thus, the peaks that appear at 2θ = 29.4° and 39.2° suggest the presence of Cu_2_O (JCPDS PDF card 65-3288) and CuO (JCPDS PDF card 89-5895) [23]. As can be seen in Figure 4, there are characteristic peaks at similar 2θ values for calcium species (CaCO_3_ and Ca(OH)_2_) or rutile. The diffraction peaks at 2θ of 18.04°, 29.69°, 34.11°, and 47.13° are ascribed to the (001), (012), and (110) planes of Ca(OH)_2_ (JCPDS PDF card 72-0156). The peaks at 2θ = 23.08 °and 29.43° show that the composition of the CaCO_3_ in the nanocomposite is the pure phase of calcite (JCPDS PDF card 47-1743). Calcium hemicarbo aluminate (Ca_4_Al_2_(OH)_12_CO_3_. 5H_2_O) was identified in the S1 and S2 diffractograms through peaks at 11.43°, 18.19°, 22.98°, 31.61°, 45.51°, and 50.94°. This compound can be result of Ca and Al interactions on TiO_2_ microparticles in alkaline conditions. Aluminum ions are commonly used as stabilizers for commercial rutile TiO_2_, and the synthesis of samples S1 and S2 was carried out in a basic environment made by the use of NaOH and Ca(OH)_2_. Similar peaks can be attributed to hydrocalumite (Ca_4_Al_2_(OH)_12_(Cl,CO_3_,OH)_2_ 4H_2_O), a mineral that can form under the same conditions, and the composition was almost the same. The diffraction peak situated at 31.64° in the S1 and S2 samples was indexed to the halite crystalline phase of NaCl.

### 3.3. X-ray Photoelectron Spectroscopy

**Figure 5:**
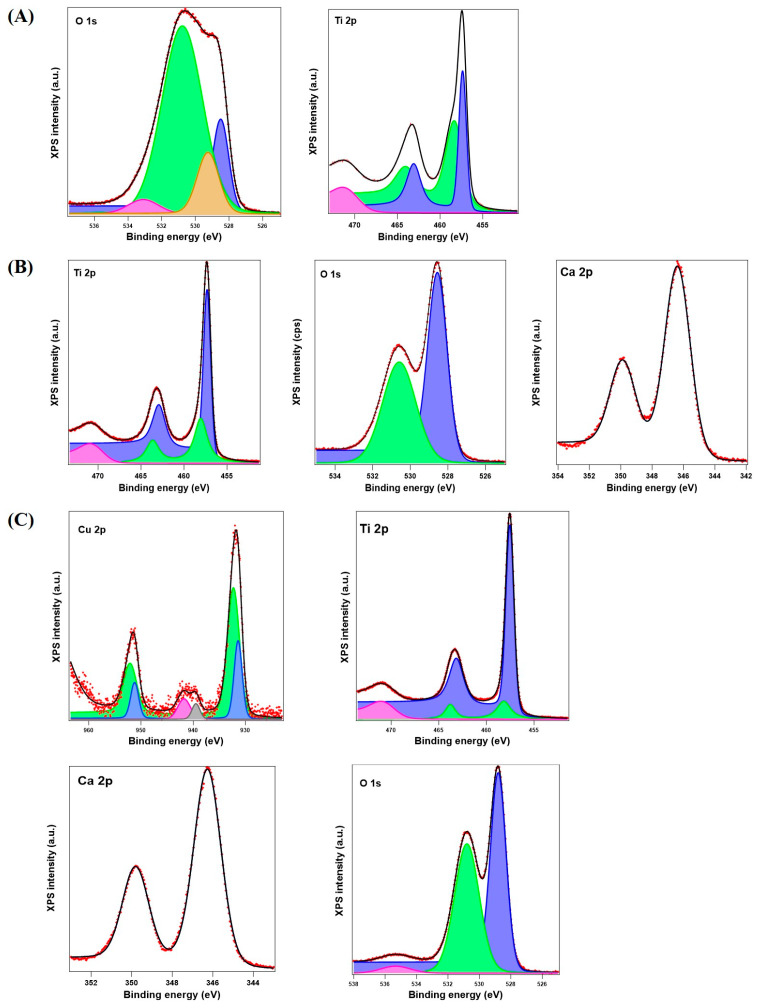
Deconvolutions of the high-resolution XPS spectra of samples: S0—(**A**), S1—(**B**), S2—(**C**). The red color line is for the observed curve, the black line is for the calculated pattern. The colored areas of the XPS represents the deconvolution results with corresponding energy values and components presented in Table 1, Table 2, Table 3, Table 4, Table 5 and Table 6.

The XPS investigation was carried out to determine the chemical composition of the sample. All the core-level spectra were deconvoluted with use of Voigt functions, singlets or doublets (Lorentzian and Gaussian widths), with a distinct inelastic background for each component (C) [24,25]. The resulted spectra are presented in Figure 5A–C.

A minimum number of components was used to obtain a convenient fit. The binding energy scale was calibrated to the C 1s standard value of 284.6 eV (measured at the beginning of the XPS spectra). The atomic composition, in Table 2, was determined by using the integral areas provided by the deconvolution procedure normalized at the atomic sensitivity factors [26]. The surface contamination with C 1s was not taken into consideration for the atomic composition.

According to the XPS results, the Ti2p spectrum is decomposed into two main peaks corresponding to Ti2p_3/2_(457.6 eV) and Ti2p_1/2_(463.3 eV), with a splitting of 5.7 eV and a small peak at ~471 eV, which indicates the presence of Ti(IV). The titanium spectra decompositions are indicated in Table 3, and they have three components (C). The main peak (Ti 2p_3/2_) has two components with a ratio C1:C2, which are 0.81 for S0, 1.67 for S1, and 1.18 for S2, respectively. The binding energies (B.E.) for the two components show that all the samples had Ti(III) oxide (C1) and Ti(IV) oxide (C2). The O 1s energies and components are presented in Table 4.

The Ca 2p spectra showed one main peak and component (~346.5 eV), which can indicate in all the samples the presence of CaO, (Table 5).

The Cu 2p spectrum is decomposed into two main peaks corresponding to Cu2p_3/2_ (931.9 eV) and Cu2p_1/2_ (951.6 eV), with a splitting of 19.7 eV. The peak at 932.3 eV, along with two satellite peaks at 939.5 and 941.8 eV, can be attributed to Cu^2+^ in CuO, while the peak at 931.4 eV can be assigned to either Cu^+^ or Cu, but taking into consideration the O 1s spectrum and its components, we can say that Cu_2_O and CuO coexisted. The ratio of C1:C2 is 0.4. The results are depicted in Table 6.

### 3.4. UV-Vis Absorption Spectra

**Figure 6:**
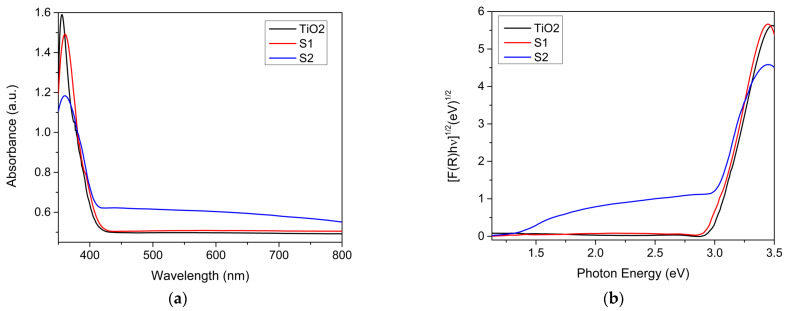
The optical absorption (**a**) and Kubelka–Munk plots (**b**) for the band gap energy calculation for TiO_2_ decorated samples.

The optical characteristics of the TiO_2_ and decorated samples were observed using UV-Vis diffuse reflectance spectroscopy and the obtained data are presented in Figure 6a. Based on the reflectance measurements, a Kubelka–Munk analysis was performed, and the resulting plots are shown in Figure 6b. For the rutile TiO_2_ and decorated samples, the obtained optical properties are depicted in Table 7.

Concerning the TiO_2_ material used in the preparation of the samples, the phase absorption edge was at a wavelength of approximately 356 nm, which was due to the transmission of electrons from the valence band of the TiO_2_ to its conduction band. This indicates that the TiO_2_ used as the synthesis material is an ultraviolet light active compound. The optical absorption spectra (Figure 6) show that the UV absorption edge is slightly sensitive to the percentage of Cu in the composition of the TiO_2_-decorated sample. Compared to the S1, the S2 sample exhibited a wide absorption band in the visible region ranging from 400 to 800 nm, making it suitable for use as an absorber material. The Kubelka–Munk plots shown in Figure 6 indicate just one band gap for the TiO_2_ and TiO_2_ decorated with Ca and two band gaps for the sample containing Cu. In sample S2, the direct band gap is due to the direct transition within the TiO_2_, while the indirect band gap corresponds to the transition within the copper. The band gap energy red shift can be attributed to sp–d exchange interactions between the band electrons and the Cu^2+^ ion substituting the Ti^4+^ lattice’s localized d electrons [27]. The Cu^2+^ introduced additional energy levels in the host band gap that were positioned below the conduction band, lowering the band gap energy of the S2 and shifting the optical sensitivity of these sample toward the visible range. This oxide mixture could display multi-band-gap semiconductor behavior, so the estimation of the band gap energy of this amorphous semiconductor could be inaccurate. The S2 sample possessed structural modifications, which may have induced intra-band-gap states that were reflected in the absorption spectrum as a broad absorption band characterized by Urbach energy [28]. The calculated values for the Urbach energy are presented in Table 7.

### 3.5. ATR FT-IR Spectroscopy

**Figure 7:**
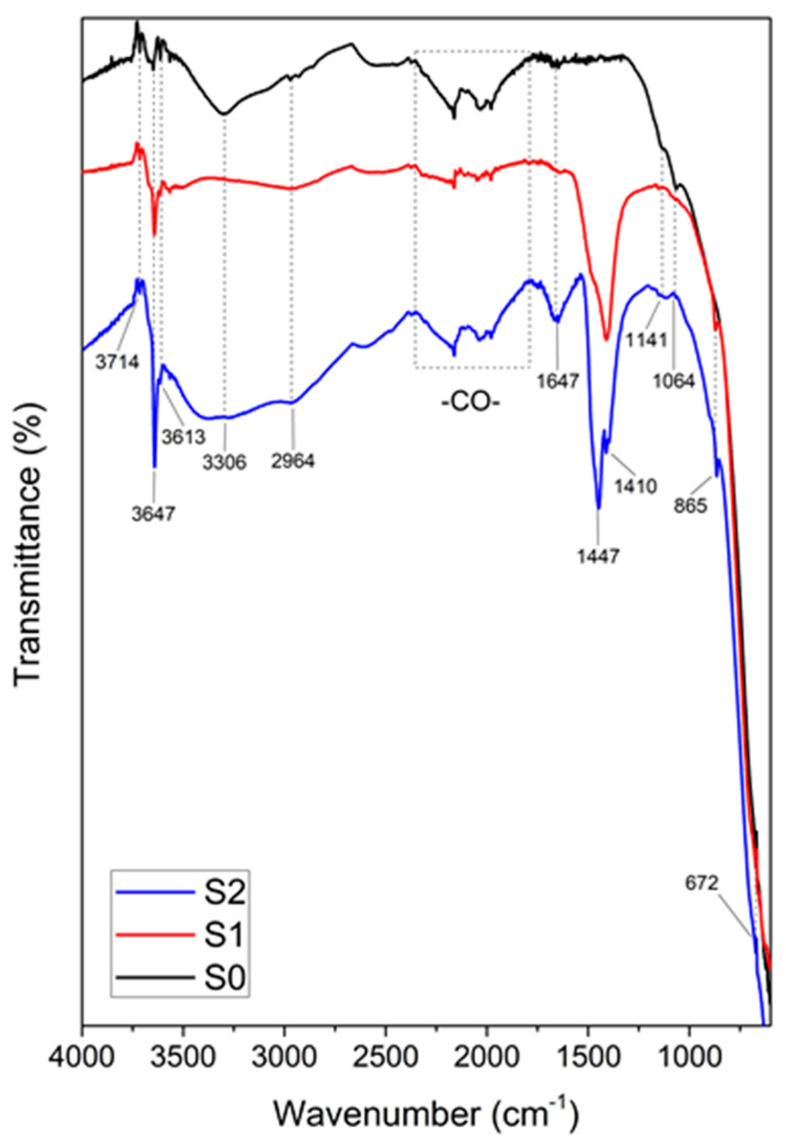
FT-IR spectra of solid powders of S1 (solid blue line) and S2 (solid red line) composite and TiO_2_ (solid black line).

The recorded ATR-FTIR spectra for samples S0, S1, and S2 are presented in Figure 7. The registered spectrum of the commercial TiO_2_ (the sample S0) reveals the adsorption bands between the reactive hydroxyl groups and the hydrophobic and hydrophilic nature of TiO_2_ [29]. This adsorption is related to the vibration and stretching modes of TiO_2_ and various oxygenated functional groups. It shows a broad band between 3800 and 3000 cm^−1^, assigned to the stretching hydroxyl group (-OH), representing the water content as moisture. The presence of a TiO_2_ coating has been reported to improve the biocompatibility of Ti, and this is attributed to the formation of the -OH bond in TiO_2_ in moist conditions [30]. However, the spectra of samples S1 and S2 show variations compared with that of S0, although it should be noted that at the wavenumbers of 3714 and 3613, the positions of the bands remain unchanged, while the peak at 3647 cm^−1^ is shifted to small wavenumbers. These bands were related to the superposition of the vibration band of the hydroxyl group and the stretching vibration of the adsorbed water molecule. Furthermore, there was a conversion of -OH sites during the synthesis process, and a selective adsorption process onto TiO_2_ surfaces could take place [31]. For samples S1 and S2, the band at 3647 cm^−1^ is due to -OH vibration in Ca(OH)_2_ specific to the portlandite phase [32]. The peak at around 2964 cm^−1^ is mainly due to the stretching vibration of the C-H bond from the absorption of the alkane groups, probably due to plastic contamination and the TiO_2_ manufacturing process. A broad band starting at 2161 cm^−1^ is due to the C-O’s presence and polyanions [33]. The adsorption C-O on the TiO_2_ in the presence of CuO under a temperature of 100 °C produced a band at 2161 cm^−1^. Species containing Cu+ ions are stabile at low temperatures [34]. Differences between the C=O groups are harder to detect; however, the peak at 1647 cm^−1^ in the samples was present in samples S1 and S2, indicating the occurrence of intermolecular hydrogen bonds between the CO group and OH in the H-OH, with a combination of phenomena related to the presence of alkali.

The peaks detected in the 1400–1600 cm^−1^ range indicate the stretching of hydroxyl and carboxyl (C=O) groups, which may have been caused by either water moisture or the titanium carboxylate. The shift in the OH vibration band towards a lower wavenumber (<1000 cm^−1^) of the metal oxide–TiO_2_ spectra compared to the TiO_2_ could have been due to the acid–base interaction of the -OH group used in synthesizing the metal oxide–TiO_2_ composite, or, possibly, to the decorating and Al_2_O_3_ impurity [35,36]. The spectra presented in Figure 7 show the presence of calcium carbonate in the S1 and S2 samples. The calcite (CaCO_3_—I phase) exhibits the typical absorption bands at 865 cm^−1^ (plane bending mode) and separated bands for the asymmetric stretch peaks split at 1396 cm^−1^ and 1410 cm^−1^, as well as at almost 1440 cm^−1^. Furthermore, the peak at 1396 cm^−1^ and 1410 cm^−1^ corresponds to the vibration of Ca in the carbonate group bound to TiO_2_. On the other hand, the absorption band at 871 cm^−1^ in S1 and 865 cm^−1^ in S2 suggest the presence of a calcite phase [37,38]. A small peak at 672 cm^−1^ corresponds to the out-of-plane bending of C-O-H and is present in all the investigated samples. When the CaO and CuO quantities were increased, the Ti-O bond vibrations shifted to smaller wavenumbers, and this revealed that the Ti and O atoms ion the Ti-O bond in the cell required less energy to vibrate with their specific wavenumber in the samples. As a result of the energy decrease, the TiO_2’_s bond length was determined to lengthen with increasing cell size. However, the peaks at lower wavenumbers are due to the increase in the metal-oxide content, together with titanium oxide [39,40]. Therefore, besides Ca^2+^, the percentage of Cu^2+^ ions was able to modulate the structural organization in the CaO-TiO_2_ structure. Copper ions can adsorb onto mineral surfaces, which facilitates carbonate adsorption through acid–base interaction in the synthesis stage. There is the possibility that the CuO added in the specific phase of the synthesis inhibit the formation of calcium carbonate crystals. Thus, in the S1 spectrum, the out-of-plane bending absorption at 871 cm^−1^ suggests the possibility of the crystallization of calcium carbonate, unlike in the S2 sample [41].

### 3.6. Density Functional Theory Calculations

**Figure 8:**
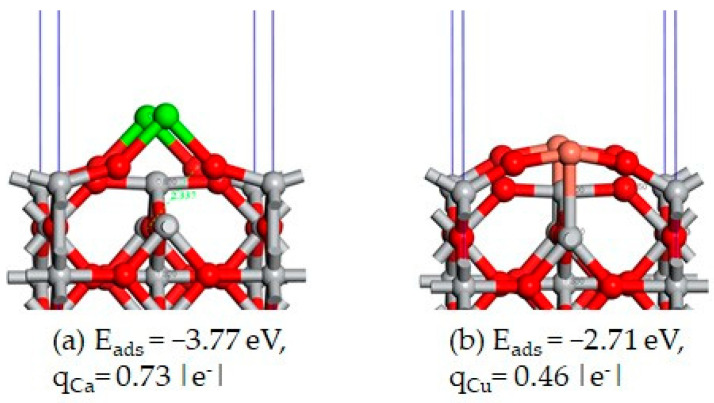
The structures of the Ca (**a**) and Cu (**b**) adsorbed in the Ti_6c_ adsorption sites on rutile TiO_2_(001) surface. The gray and red spheres represent the titanium and oxygen atoms, respectively, and the green and brown spheres represent the adsorbed metal atoms M = Ca and Cu, respectively. The adsorption energy and the charges of the adsorbed atoms are given below the corresponding figures.

The top layer has a slight contraction of 0.19 Å and a slight rumpling of 0.11 Å, and the oxygen/titanium atoms moved outwards from/inward and toward the surface (see Figure 2c). The slab that consists in seven atomic layers is large enough to ensure the convergence of the Mulliken charges on the atoms located in the interior layers (the two layers below each of the two faces of the slab) towards the values corresponding to the bulk case, q_Ti_ = 1.28 and q_O_ = −0.64 |e^−^|. In cases of the adsorption of a metal atom M = Ca or Cu over the Ti_6c_, the atom forms chemical bonds with the two neighboring oxygen atoms, O_2c_. The two O_2c_ atoms moved slightly outward from the surface and broke their chemical bonds with the Ti_6c_ atom that was located below the adsorbate. The Ca atom preferred to stay out of the surface, but Cu atom was almost incorporated into the top layer of the surface, making a chemical bond with the Ti_6c_ (see Figure 8a,b).

**Figure 9:**
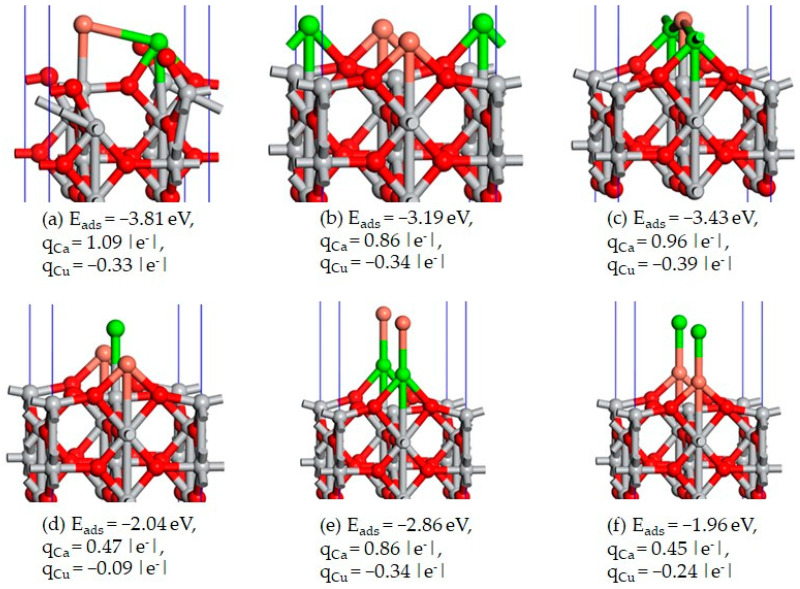
The structures of the co-adsorbed Ca and Cu on the Ti_4c_ and Ti_6c_ sites of rutile TiO_2_(001) surface. The color significations are the same as in Figure 8. The adsorption energy (E_ads_) and the charges of the adsorbed atoms (q_Ca_ and q_Cu_) are given below the corresponding figures.

The adsorption of the second metal atom of the different element type near the first adsorbed atom is more favorable than the adsorption of only one metal atom M = Ca or Cu. Through the adsorption of this second metal atom, the broken bond between O_2c_ and Ti_6c_ is restored. The most favorable co-adsorption is Ca/Ti_6c_–Cu/Ti_4c_, with E_ads_ = −3.81 eV (see Figure 9a). The inverse adsorption Cu/Ti_6c_–Ca/Ti_4c_ is highly unfavorable, with E_ads_ = −2.84 eV. The adsorption of the second metal atom on top of the first adsorbed atom is also stable, the preferred co-adsorption site being Ti_6c_ (see Figure 9e,f).

**Figure 10:**
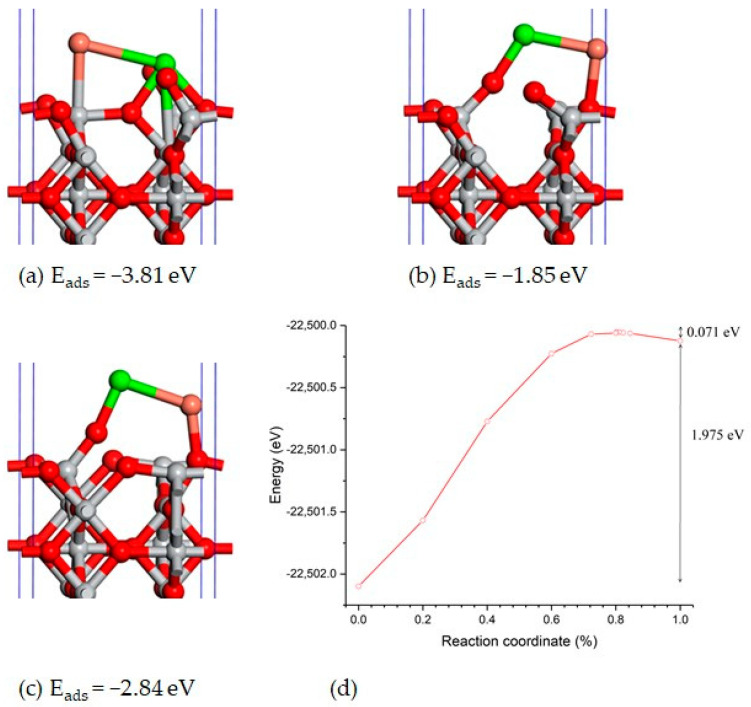
The structures of the adsorptions in the initial (**a**), transition state (**b**), and final configuration (**c**) of the transformation from Ca/Ti4c-Cu/Ti6c to Cu/Ti4c-Ca/Ti6c, and the energy evolution along the transformation path (**d**). The color significations of the atoms are the same as in Figure 8.

Diffusion on a rutile surface can be activated by thermal excitation; at room temperature (T = 300 K), an energy of k_B_T = 0.025 eV is necessary, where k_B_ is the Boltzmann constant. By identifying the corresponding transition state (see Figure 10b), the activation energy of the transformation from the most stable co-adsorption configuration Ca/Ti_6c_–Cu/Ti_4c_ (see Figure 10a) to the inverse configuration Cu/Ti_6c_–Ca/Ti_4c_ (see Figure 10c) was computed.

The transformation consists in the translation of the diatom Ca-Cu along the direction of the triplet, O_2c_-Ti_4c_-O_2c_ (see Figure 10a–c), where the periodicity of the system has to be considered. This transformation requires a much higher activation energy, of 1.975 eV, but the inverse transformation only requires 0.071 eV. Such calculations indicate that at equilibrium, most of the adsorbed metals, Ca and Cu, preferentially occupy the sites Ti_6c_. The obtained calculation corresponds to a very high concentration and a regular arrangement of the co-adsorbed metal atoms. We are aware that a larger supercell in the plane of the surface is necessary to avoid the interaction between the co-adsorbed metal atoms and their periodic images. Compared to the Mulliken-computed atomic charges (q_O_ = −0.64 |e^−^| and q_Ti_ = 1.28 |e^−^|) for the oxygen and titanium atoms from the bulk of the rutile TiO_2_, the charges of the atoms from the first and the second layers of the surfaces are q_O_ = −0.66 |e^−^|, q_Ti_ = 1.24 |e^−^|, q_O_ = −0.62 |e^−^|, and q_Ti_ = 1.31 |e^−^|, respectively, indicating a charge redistribution between the atoms from these two surface layers. The charges of the atoms from the below layers have the same values as those from the bulk. Therefore, these internal layers are considered to behave as the bulk layers. When a metal atom M = Ca or Cu adsorbs on surface, it loses a fraction of an electron, such that the corresponding charges are q_Ca_ = 0.73 |e^−^| and q_Cu_ = 0.46 |e^−^|. When both Ca and Cu adsorb on the surface of TiO_2_, the Cu atom gains a fraction of an electron, mainly from Ca atoms, being negatively charged with values between −0.39 and −0.09 |e^−^|.

### 3.7. Antibacterial Properties

#### 3.7.1. Biological Test

**Figure 11:**
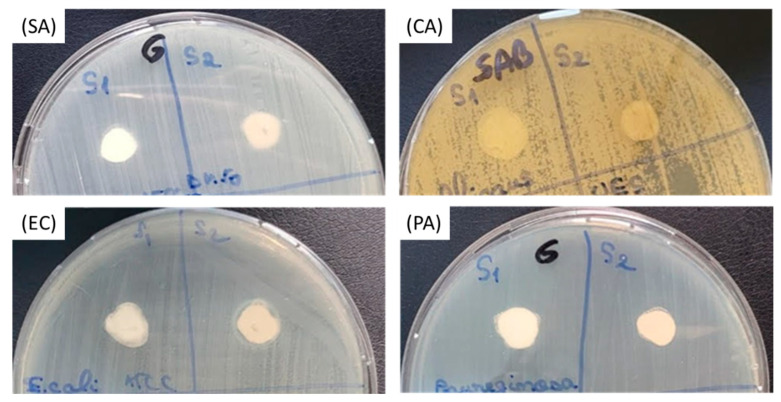
Aspect of the Gram-positive (*S. aureus* ATCC 25923—(SA) and *C. albicans* ATCC 10231—(CA)) and Gram-negative (*E. coli* ATCC 25922—(EC) and *P. aeruginosa* ATCC 27853—(PA)) microbial cultures, after placing the pigment drops on the surface of the culture medium.

The qualitative results show that no inhibition zone extended over the contact area of the pigment drops with the microbial cultures for all the tested strains (Figure 11). In this condition, the diameters of the inhibition zone were considered to be the same as the diameters of the pigment drops (Table 8), in concordance with the CLSI standard, the tested concentration of the two pigments being 100 mg/mL.

The quantitative evaluation of the antimicrobial efficiency was performed by determining the minimal inhibitory concentrations (MICs) of pigment suspensions after 20 h of contact with the microbial cells, in a liquid medium. The values, expressed in mg/mL, are included in Table 9. Compared to the qualitative tests, testing the antimicrobial activity in a liquid medium potentiates the inhibitory effect of the two pigment samples, with the pigment concentration value that ensures the inhibition of microbial growth and multiplication being between 5 and 1.25 mg/mL.

**Figure 12:**
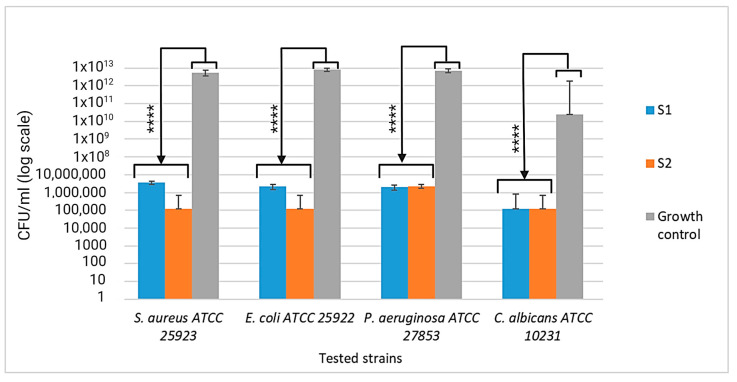
Graphical representation of the log10 values of colony-forming units (CFU)/mL representing the viable cell number, at MIC. The significant inhibition of multiplication at the MIC value can be observed for all sensitive microbial strains. The results were compared using two-way ANOVA and Dunnett’s multiple comparisons tests. The results were considered statistically significant (**** *p* < 0.0001).

The inhibitory effect of the tested pigments at MIC values was confirmed by determining the CFU/mL in the corresponding MIC well. The results were statistically processed and integrated into Figure 12. According to the CLSI standard, the inhibitory effect is significant at a difference of at least four logarithmic units, compared to the growth control, which can be observed for all the tested microbial strains (Figure 12).

The biological activity of the TiO_2_ -based composites demonstrated by the antimicrobial tests in the liquid medium can be explained based on the interactions between the negative electrical charge of the microbial cell wall and the positively charged composite layers supported on the TiO_2_ microparticles. Gram-positive and Gram-negative bacteria have differences in cell wall structure, with the cell walls of Gram-negative bacteria having an additional outer membrane, in which negatively charged lipopolysaccharide molecules are embedded. At the same time, the cell walls of Gram-positive bacteria possess only an inner membrane, a representative layer of peptidoglycan crossed by negatively charged teichoic acids.

#### 3.7.2. Physicochemical Microbial Cell Surface Characterization

The ZP values as a function of time (7 h) for *Staphylococcus aureus* (SA), *Pseudomonas aeruginosa* (PA), *Candida albicans* (CA), and *Escherichia coli* (EC) microorganisms before and during exposure to S1 and S2 composite dispersions were measured. The pigment S1 and S2 dispersions were prepared in PBS with a concentration of 0.01 mg/mL by using a sonication probe. The average ZP values were −29.18 mV for S1 and −25.66 mV for S2, respectively, and these values were used in the data processing of the bacterial-pigment systems (SA-S1, SA-S2, PA-S1, PA-S2, CA-S1, CA-S2, EC-S1, EC-S2).

**Figure 13:**
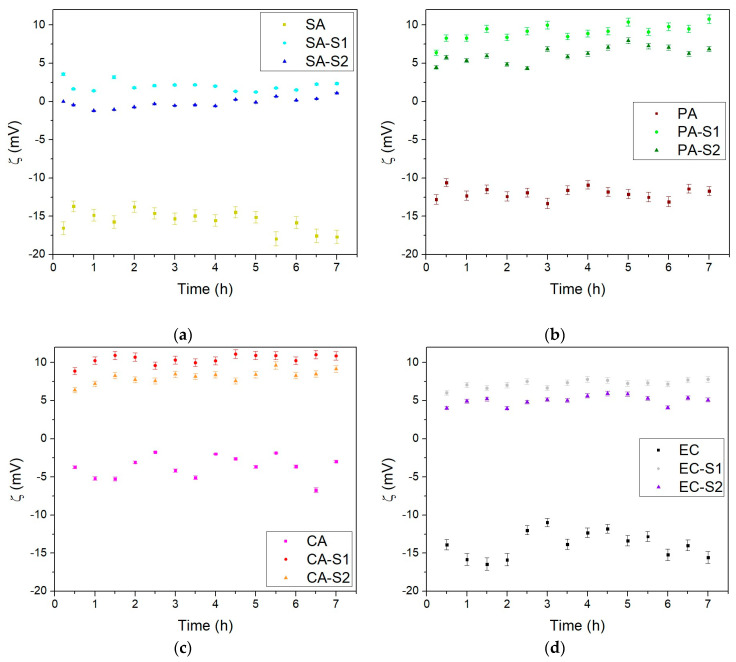
Zeta potential as a function of time (7 h) for (**a**) *S. aureus* (SA) before and during exposure to S1 (SA-S1) and S2 (SA-S2) and (**b**) *P. aeruginosa* (PA) before and during exposure to S1 (PA-S1) and S2 (PA-S2). (**c**) *C. albicans* (CA) before and during exposure to S1 (CA-S1) and S2 (CA-S2) and (**d**) *E. coli* (EC) before and during exposure to S1 (EC-S1) and S2 (EC-S2).

In Figure 13a–d, it can be observed that the zeta potential was mildly negative for the SA, PA, CA, and EC pathogen surfaces. The measured negative value of the zeta potential was ascribed to the existence of specific functional groups in the composition of the outer layer of the bacterial membrane. It can be seen for the SA and EC cells that the ZP became more negative with aging. In contrast, the PA and CS cells showed a stationary phase of development, with the ZP values becoming less negative. After the exposure to S1 and S2, the ZP values showed that the physiological condition of pathogens was modified beyond the positive values of the ZP, suggesting possible damage to the cell structure. Furthermore, the ZP values’ order depended on the bacterial species, and exposure to S1 seems to be more harmful than exposure to S2 for all the studied pathogens.

As can be observed in Figure 13, the zeta potential values of the S1 and S2 samples and the TiO_2_ used in the synthesis had negative values in the range of −30 mV to −25 mV. For the S1 and S2 samples dispersed in the PBS solution, under-coordinated oxygen and Ca and Cu ions at the TiO_2_ surface reacted with water molecules to form surface hydroxyl functional groups. The dissociation of surface hydroxyl groups results in the formation of a net charge at the particle surface (TiO_2_ decorated with Cu and/or Ca). Consequently, the bulk solution will become a proton acceptor and leave the particle surface to become negatively charged. Thus, in buffer conditions (at pH 7), it was observed that the S1 and S2 samples exhibited a negative surface charge, as evidenced.

#### 3.7.3. Morphological Characterization of the Bacterial Cells over the Pigment Surfaces

**Figure 14:**
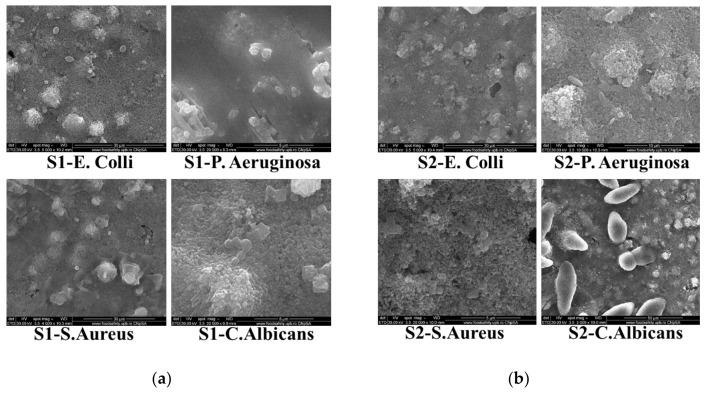
Representative SEM images of *E. coli*, *P. Aeruginosa*, *S. Aureus*, and *C. Albicans* bacterial cells after exposure to S1 composite (**a**) and S2 composite (**b**).

Microbial suspensions at a density of 1.5 × 10^−8^ CFU/mL were prepared from the SA, PA, CA, and EC microbial cultures, then 100 μL of each sample was spotted over the deposited S1 and S2 pigments. After equal diffusion of the compound in the medium, at room temperature, the prepared plates were incubated for 2 h at 37 °C under darkness conditions. After fixation and dehydration, the surface morphologies of the SA, PA, EC, and CA microorganisms in the presence of the pigments were analyzed using SEM, and the obtained micrographs are presented in Figure 14a, for composite S1, and Figure 14b, for composite S2. The contours of the considered organisms in the SEM images exhibit irregularities, including hollows or bumps. These irregularities might be a consequence of cellular degeneration after exposure to pigments. Further, the loss of shape for the SA and EC appears more shrunken or distorted compared to that for the PA and CA.

## 4. Discussion

### 4.1. The Impact of Ca and Cu on Surface Modification of Rutile

It is well known that the excitation of TiO_2_ for photocatalytic reactions is specifically triggered by UV-A light in the range of 370–380 nm [42]. This represents a limitation of the antibacterial capacity of the pigment in low-light or dark conditions. Consequently, the goal of this research was to develop an improved antimicrobial composite based on Ca- and Cu-decorated TiO_2_ microparticles to overcome the limitations of existing materials by improving antimicrobial efficiency. Moreover, the studied pigments must serve as an eco-friendly coating solution with lasting effectiveness. One of the main reasons for the preference for rutile in S1 and S2 pigment production is its higher resistance to UV light compared to anatase. Rutile titanium dioxide is more resistant to UV light, making it suitable for use in outdoor conditions. This property makes rutile a preferred choice for paints that will be exposed to sunlight and other harsh environmental conditions. Compared to anatase, rutile has a higher refractive index, which provides better opacity and brightness in paints. The crystal structure of rutile allows for improved light scattering, enhancing the paint’s covering power. Furthermore, the particle size of titanium dioxide also plays a role in its application in paints. Studies have shown that the optimum particle size for pigmentary titanium dioxide is around 250 nm [43]. The commercial rutile used in our pigments consists in round particles with diameters of 100 nm to 200 nm (Figure 3a).

According to the XPS data (Figure 5), the rutile used in the S1 and S2 samples’ preparation presents Ti3+ sites, which have distinct electronic properties that can influence the surface chemistry and reactivity of rutile [44]. In an extreme basic environment, hydroxylated rutile undergoes deprotonation, leading to the formation of Ti-O-species. The activation of TiO_2_ surfaces in the presence of alkali hydroxides involves the formation of a layer with an open porosity, which acts as a scaffold for the capture on the TiO_2_ surface of other chemical species existing in the solution. Thus, there is the possibility of a Ti–O–Me structure type (Me = Ca, Na, Cu) resulting through electrostatic bonds that subsequently contribute to the incorporation of additional hydroxyl groups [45,46,47]. In the XRD and FT-IR spectra of samples S1 and S2, the presence of portlandite was proven, and it can be assumed that a pozzolanic-like reaction could take place between concentrate alkaline hydroxides and aluminous materials with TiO_2_ [48]. Thus, Ca ions released from Ca(OH)_2_ to form Ti-O-Ca hydrate structures with a duration of exposure to environmental CO_2_ will grow calcium–aluminate–hydrate phases [49]. Therefore, the presence of hydrocalumite in samples S1 and S2 was demonstrated. Its presence could be attributed to the carbonation and dehydration processes, which increased the concentration of metallic cations such as calcium and aluminum, resulting in an excess of positive electrical charges that had to be balanced by negatively charged anions. This may contribute to the formation of minerals exhibiting an asymmetric arrangement of atoms such as hydrocalumite.

Furthermore, Ca(OH)_2_ crystals are precipitated after mixing supersaturated aqueous solutions of calcium chloride (CaCl_2_) and sodium hydroxide (NaOH). After the reaction, different amounts of Ca(OH)_2_ and NaOH will remain in solution. The resulting hydroxides react with atmospheric carbon dioxide to produce carbonates. Additionally, the presence of Na and Ca ions on rutile surfaces produces active sites for water and CO_2_ adsorption. NaOH and Ca(OH)_2_ are basic enough to retain water and CO_2_ through O sites, and a possible way to retain CO_2_ is through a tridentate carbonate-like structure [50]. As our recorded data reveal, carbonate is present through the calcite phase. The particles of decorated TiO_2_ with hexagonal plate-like carbonate crystals can be seen in the micrographs presented in Figure 3.

In addition, the presence of CaCO_3_ may affect the optical properties of TiO_2_, potentially altering its performance in applications requiring specific optical characteristics [6]. Therefore, the photocatalytic properties of TiO_2_ are altered in the presence of carbonates, and to correct this shortcoming, Cu was added in a synthesis step. In a previous study, the effectiveness of the Cu content in the pigment composition was evaluated. It has been established that it is efficient and advantageous to use 2% Cu in the preparation of the pigment [51].

In sample S2, the presence of Cu(I), Cu(II), and Cu^0^ was demonstrated. The CuO bonded on the TiO_2_ surface was strongly reduced through electronic gain, as the DFT results suggest (Figure 11). In alkali environments under soft thermal treatment, Cu(II) can participate in the Cu(II)/Cu(I) redox cycle, which leads to the formation of hydroxides, or even cuprates [52]. Moreover, such species are present on TiO_2_ surfaces. The stability of Cu ions depends on the hydration energy when they bond water molecules; thus, Cu(II) is more stable in aqueous solutions than Cu(I) [53]. Moreover, due to their large charge density, Cu(II) ions form much stronger bonds with TiO_2_ surfaces, releasing more energy. CuO can act as an electron trap, capturing and reducing the concentration of free electrons available for synthesis reactions. Consequently, the generation of Cu(I) species on the TiO_2_ surface plays a significant role in facilitating photo-induced charge transfer, which is crucial for efficient photocatalysis [15]. In the S1 and S2 samples, the presence of alkaline atoms did not modify the band gap of the TiO_2_, and this indicates that only the surface layer of the TiO_2_ was implied in the interactions. The modification of rutile TiO_2_ with Ca does not induce a shift in light absorption. A red shift in light absorption was induced when Cu(I) was present in the samples (Figure 6).

Overall, the S1 and S2 samples demonstrated strong oxide–CO_2_ interactions, which play a crucial role in facilitating the capture and storage of CO_2_ technologies. Moisture cycling helps regulate the moisture content in composites, ensuring that they can continue capturing CO_2_ efficiently. The management of moisture levels and the capacity of CO_2_ absorption could be future points in our research.

### 4.2. The Influence of Composite Properties on Bacterial Strain

Samples S1 and S2 were designed to target and tailor an antimicrobial strategy in low-light and dark conditions. These Ca- and Cu-decorated TiO_2_ microparticles exhibit strong basic properties, and also photocatalytic activity, through the presence of TiO_2_. Photocatalysis and alkaline environments are both effective methods for killing bacteria, but the strength of their antimicrobial effects depends on various factors and conditions. It is well known that TiO_2_ exposure to UV or visible light generates hydroxyl radicals and hydrogen peroxide, which can degrade bacterial cell walls and membranes, leading to cell death. This process has been shown to be capable of killing a wide range of bacteria, fungi, viruses, and other microorganisms [54,55]. It is worth noting that the effectiveness of photocatalysis mainly depends on the intensity and duration of light exposure, and this situation becomes limiting when the pigment is applied in dark environments. Alkaline environments can have a detrimental effect on bacteria, even in dark conditions, with respect to certain concentrations and durations of exposure. Additionally, alkaline environments may not have the same broad-spectrum antimicrobial activity as photocatalysis [56]. For instance, the adaptation of alkaliphilic bacteria to survive and reproduce in alkaline environments (pH of 12–13) has been proven [57].

The composites S1 and S2 provided alkaline environments in the presence of moisture. Furthermore, the dry powders of S1 and S2 are hygroscopic and very reactive through hydroxyl groups. The measured pH values varied for the S2 sample, from a pH of 12.5 in low moisture to a pH of 8 for a concentration of 10 μg/L. The qualitative and quantitative biological tests demonstrated the antibacterial efficiency of composites S1 and S2. Moreover, the zeta potential values showed the lack of antibacterial activity in the presence of a very low concentration of the composite (10 μg/L composite in PBS). Our studies have shown that bacterial growth and survival were inhibited in an alkaline environment when exposure took place in room light and dark conditions. This occurs when the high pH disrupts the bacterial cell membrane and proteins, leading to their structural changes and loss of function. Additionally, alkaline conditions can interfere with essential cellular processes, ultimately causing microbial cell death for both Gram-positive and Gram-negative bacteria. It is most likely that the implied mechanisms involve, to some extent, separately or combined, the following processes: membrane depolarization, reactive oxygen species generation, apoptosis-like cell death, and pH homeostasis disruption.

## 5. Conclusions

In this work, composite layers with Ca- and Cu-decorated TiO_2_ microparticles were physicochemically characterized, and quantum chemical calculations were performed to evaluate the interaction of the calcium and the copper with the rutile surfaces.

The behavior of the four microbial strains, *Staphylococcus aureus*, *Escherichia coli, Pseudomonas aeruginosa*, and *Candida albicans*, in the presence of Ca- and Cu-decorated TiO_2_ microparticles, was studied.

The qualitative tests and the quantitative evaluation of the antimicrobial efficiency revealed the inhibition of microbial growth. The zeta potential data indicate that the physiological condition of the investigated microbial strains was strongly affected, and the recorded SEM images show a damaged microbial cell surface, in the presence of composites.

Based on the obtained results, the antimicrobial properties of the new synthesized Ca- and Cu-decorated TiO_2_ microparticle samples were proven; moreover, it was demonstrated that their effectiveness is potentiated in the presence of humidity. There were no special lighting conditions to activate the antimicrobial properties of these pigments. They were maintained in the dark or under normal lighting conditions of a room.

Considering the structure and simulation data, it was discussed that the antimicrobial efficiency of composites S1 and S2 was possibly due to the presence of Na and Ca carbonate components and the charge transfer from Ca to Cu when these atoms were absorbed on the TiO_2_ surfaces.

## Figures and Tables

**Table 1:** Percent of the materials used in the synthesis.

Sample Name	Abbrevion	Ca (%)	Cu (%)	Observations
TiO_2_	S0	0	0	Commercial rutile
TiO_2_ + Ca	S1	20	0	-
TiO_2_ + Ca + Cu	S2	20	2	-

**Table 2:** Composition of the materials used in the investigations.

Sample	Ti 2p (%)	O 1s (%)	Ca 2p (%)	Cu 2p (%)
S0	84.01	15.99	-	-
S1	23.15	69.22	7.63	-
S2	30.68	61.36	7.33	0.63

**Table 3:** The components of each sample with their binding energies (B.E.) and the contribution of each component (C) to the total intensity of Ti 2p.

Sample	S0		S1		S2	
Component	B.E. (eV)	C (%)	B.E. (eV)	C (%)	B.E. (eV)	C (%)
C1	457.4	40.73	457.3	56.68	457.5	50.01
C2	458.4	50.18	457.9	33.98	458.3	42.66
C3	470.8	9.09	470.6	9.15	470.7	7.33

**Table 4:** The components of each sample with their binding energies and the contribution of each component to the total intensity of O1s.

Sample	S0		S1		S2	
Component	B.E. (eV)	C (%)	B.E. (eV)	C (%)	B.E. (eV)	C (%)
C1	528.5	13.37	528.6	51.37	528.8	56.55
C2	529.3	12.33	-	-	-	-
C3	530.8	70.29	530.6	48.63	530.8	40.4
C4	533.1	4.01	-	-	535.3	3.05

**Table 5:** The components of each sample with their binding energies and the contribution of each component to the total intensity of Ca 2p.

Sample	S1		S2	
Component	B.E. (eV)	C (%)	B.E. (eV)	C (%)
C1	346.4	100	346.3	100

**Table 6:** The components of the S2 sample with their binding energies and the contribution of each component to the total intensity of Cu 2p.

Sample	S2	
Component	B.E. (eV)	C (%)
C1	931.4	25.47
C2	932.3	64.18
C3	939.5	3.36
C4	941.8	6.99
C5	964.4	3.31

**Table 7:** Absorption edges and the calculated value of optical band gap (eV) and Urbach energy (eV) of the investigated samples.

Sample	Absorption Edge (nm)	Band Gap (eV)	Urbach Energy (eV)	Band Gap (eV)	Urbach Energy (eV)
TiO_2_	356	3.01	0.41	-	-
S1	358	2.92	0.41	-	-
S2	359	2.89	0.47	1.25	2.50

**Table 8:** Inhibition zone diameter values (mm).

Microbial Strains	Inhibition Zone Diameters (mm)	
	S1	S2
*S. aureus* ATCC 25923	10	10
*E. coli* ATCC 25922	10	10
*P. aeruginosa* ATCC 27853	10	10
*C. albicans* ATCC 10231	10	10

**Table 9:** Minimal inhibitory concentration values for the two pigment samples (mg/mL).

Tested Sample/Tested Strains	Minimal Inhibitory Concentration (mg/mL)			
	*S. aureus* ATCC 25923	*E. coli* ATCC 25922	*P. aeruginosa* ATCC 27853	*C. albicans* ATCC 10231
S1	5	5	2.5	1.25
S2	2.5	2.5	2.5	1.25

## Data Availability

The original contributions presented in the study are included in the article, further inquiries can be directed to the corresponding authors.
